# On temporal evolution of the response of EBT4 radiochromic films

**DOI:** 10.1002/acm2.70514

**Published:** 2026-02-19

**Authors:** Arash Darafsheh, Ananya Parimi, Megha Goddu, Hamid Ghaznavi, Aditi Purushothaman, S. Murty Goddu

**Affiliations:** ^1^ Department of Radiation Oncology WashU Medicine St. Louis Missouri USA

**Keywords:** dosimetry, EBT4, film darkening, optical density, polymerization, radiochromic film

## Abstract

**Background:**

Radiochromic film dosimeters are commonly used in radiation oncology in both clinical and research settings to measure the radiation dose with high spatial resolution over a two‐dimensional area. Radiation‐induced solid‐state polymerization occurring in films leads to a change in their opacity. It is critical to evaluate the temporal stability of the response of new film models to ensure an appropriate post‐irradiation time is selected for film scanning.

**Purpose:**

To investigate the time‐dependent growth of optical density (OD) in the recently released EBT4 radiochromic films.

**Methods:**

EBT4 film samples were irradiated at 0.125, 0.25, 0.5, 1, 2, 3, 4, 5, 6, 7, 8, 9, 10, 12, and 15 Gy dose levels using a 6 MV X‐ray beam produced by a clinical linear accelerator. Two film samples were irradiated at each dose level. The films were repeatedly scanned using a flatbed scanner upon irradiation and at various time intervals for approximately 2 months (1325 h). The net OD was measured, and the films’ sensitivity and calibration curves were obtained at several time points.

**Results:**

The net OD grows with time in all color channels, the rate of which reduces with dose. Red channel OD increased by ∼8%–30% over the studied time period for films irradiated at 15‐1 Gy. A similar trend was observed in the green channel with slightly higher (∼1%–2%) growth rates compared to the red channel. Films irradiated at > 1 Gy dose reached over ∼90% of their final net OD within the first 12 h post‐irradiation, followed by a gradual growth over the following weeks. The OD‐per‐Gy decreased with dose and increased with elapsed time post‐irradiation.

**Conclusion:**

Since the response of the films continues to develop with time, to adhere to good practice of film dosimetry, the calibration and test films must be read at the same time interval post‐irradiation, otherwise significant errors in dose measurement can occur. Since the temporal dynamics of EBT4 and EBT3 films were found to be similar, both models can be treated similarly in terms of the optimum post‐irradiation wait‐time prior to film readout.

## 1 INTRODUCTION

Radiochromic films (RCFs) are ubiquitous in radiation oncology clinical and research settings to measure the two‐dimensional (2D) dose distribution with high spatial resolution.^1^, ^2^, ^3^ The most widely used brand of RCFs in radiation oncology applications is GafChromic™ film manufactured by Ashland Inc. (Bridgewater, NJ, USA). According to the manufacturer, different GafChromic™ film models are available for various dose ranges: EBT3 (0.2–10 Gy), EBT‐XD (0.4–40 Gy), and MD‐V3 (up to 100 Gy).^4^ However, several studies have investigated the performance of these films over dose ranges that extend beyond the manufacturer‐recommended intervals; for example, EBT3 has been evaluated up to 50 Gy^5^ and EBT‐XD has been studied up to 50–60 Gy.^6^, ^7^

Recently, EBT4 model was introduced as a modified version of the EBT3 model which employs a modified active‐layer/substrate architecture that improves the signal‐to‐noise ratio,^8^, ^9^ reduces orientation dependency^10^ and lateral response artifact,^11^ and results in modestly lower sensitivity (optical density‐per‐Gy)^2^ compared to the EBT3 model. Due to their novelty, it is important to evaluate their characteristics in comparison to their predecessor EBT3 model. In terms of energy dependency^12^, ^13^ and spectral characteristics,^14^ it has been verified that the EBT4 model behaves similarly to the EBT3 model. The literature on EBT4 films indicates that while the basic EBT4 film architecture is similar to EBT3, EBT4 displays measurable differences in channel sensitivity and orientation‐related artifacts that justify explicit model‐specific characterization for film dosimetry.

Another important characteristic of RCFs that needs to be evaluated before use is the temporal stability of the film's response (e.g., OD) to radiation. Initiation, polymerization, and termination are three steps that occur during the response of RCFs to radiation. Diacetylene monomer crystals in the active layer of the RCFs undergo radiation‐induced solid‐state polymerization due to the formation of reactive intermediate energetic radicals (for a review, see Devic et al.^15^). Polymerization reactions are covalently crosslinked by ionizing radiation, the degree of which depends on the absorbed dose. The dose‐dependent film opacity as a result of radiation‐induced polymerization is quantified by OD in order to perform quantitative film dosimetry.^16^ However, it has been demonstrated that the OD grows with post‐irradiation time at different rates in various RCF models: now obsolete DM‐1260,^17^ MD‐55,^18^ MD‐55‐2,^19^, ^20^ EBT,^21^ and EBT2^22^ models, as well as currently available EBT3,^23^, ^24^, ^25^, ^26^, ^27^ EBT‐XD,^28^ EBT4,^8^ and OC‐1 (OrthoChrome Inc.)^29^ models. As such, it is vital to understand the temporal stability of OD in each new film model to ensure that a stable post‐irradiation time is selected for film scanning. Given that EBT4 is a recently released model and that the film response to radiation is model‐dependent, in this work, we investigate the post‐irradiation growth of net OD in EBT4 films over an extended period (∼1325 h, ∼2 months) across a range of clinically relevant doses (1–15 Gy). We derive time‐dependent calibration curves to evaluate the influence of temporal dynamics of the films on their dosimetric accuracy to provide practical readout guidance for accurate film dosimetry.

## 2 METHODS

**FIGURE 1:**
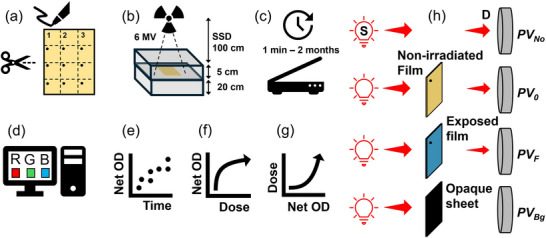
(a) Film preparation: EBT4 films were cut into 3.8 cm × 3.8 cm pieces. Top left corner in all pieces was permanently marked to ensure that the same orientation is maintained during pre‐ and post‐irradiation scanning of all film samples. (b) Films, placed at 5 cm depth in solid water phantom, were irradiated with a 6 MV beam produced by a linear accelerator (linac) at various dose levels (0.125–15 Gy). (c) Using a flatbed scanner, film samples were repeatedly scanned upon irradiation until approximately 2 months (∼1350 h) after irradiation at various time intervals. (d) Using Python, pixel values were obtained for the red, green, and blue channels. (e) Net optical density as a function of time for each dose level was obtained. (f) In order to calculate the film sensitivity, response curves demonstrating *Net OD* as a function of dose were obtained. (g) Calibration curves were obtained at several selected time intervals post‐irradiation (0.17 h (10 min), 1 h, 2 h, 6 h, 12 h, 24 h, 48 h, 120 h (5 days), 240 h (10 days), 730 h (∼1 month), and 1325 h (∼ 2 months)). (h) Schematic illustration of the geometry of the experimental setup to measure the optical density of a film sample. *PV_NO_*, *PV_0_*, and *PV_F_* represent the pixel value of the digital image in the absence of the film sample, for the nonirradiated film, and for the irradiated film, respectively. *PV_Bg_* represents any background signal (e.g., dark current) when a completely opaque sheet is placed on the scanner's bed as the sample. S: Light source, D: Detector (scanner in this case). Adapted from Darafsheh and Ghaznavi.^2^

Figure 1(a–g) describes the experimental procedure. Sheets of EBT4 radiochromic films (Lot# 07052201) were cut into 3.8 cm × 3.8 cm pieces using a paper cutter guillotine to avoid mechanical stress or delamination of the film layers. To ensure that the film orientation is kept the same during the scanning, the top left corner of each film was marked. Films were handled according to the recommendations of the AAPM's task group (TG) report 235.^30^

Film samples were irradiated at 0.125, 0.25, 0.5, 1, 2, 3, 4, 5, 6, 7, 8, 9, 10, 12, and 15 Gy dose levels using a 6 MV X‐ray beam produced by a clinical linear accelerator (TrueBeam™, Varian Medical Systems). Two films samples (referred to as Set 1 and Set 2) were studied at each dose level. The linear accelerator was calibrated in accordance with the AAPM's TG‐51 protocol^31^ to deliver 1 cGy per monitor unit (MU) at the depth of maximum dose (1.5 cm) in a phantom, using a 100 cm source‐to‐surface distance (SSD) and a 10 cm × 10 cm field size at the surface. As illustrated in Figure 1(b), the films were positioned at a depth of 5 cm in a phantom with an additional 20 cm of phantom material placed beneath them to ensure full backscatter conditions. The SSD was 100 cm. The required monitor unit (MU) at each dose level (D) was calculated through the percentage‐depth dose (PDD) at 5 cm depth (MU = D/PDD(5 cm)), which resulted in 116.1 MU to deliver 1 Gy to the film.

Upon irradiation, the films were scanned with a flatbed scanner (Expression 10000XL, Epson America, Inc.). The scanned images were saved as 48‐bits tagged image file format (TIFF) files at 300 dpi. All scanner automatic image‐processing functions (e.g., color correction and automatic exposure) were disabled for all scans. Films were scanned repeatedly at various post‐irradiation times for approximately 2 months. Selected time points of 0.17 h (10 min), 1 h, 2 h, 6 h, 12 h, 24 h, 48 h, 120 h (5 days), 240 h (10 days), 730 h (∼1 month), and 1325 h (∼ 2 months) were used for quantitative analysis of the growth rate in film darkening.

In order to perform an overall comparison between the EBT4 and EBT3 model, EBT3 film samples (Lot# 03232302) were irradiated at 5 Gy and 8 Gy and scanned repeatedly for the first 12 h after irradiation, and then every 24 h over a 5‐day period.

In order to measure the OD from each scanned image, Equation (1) was used. For each color channel, the mean pixel value over a 100 pixel by 100 pixel (∼8.5 × 8.5 mm^2^) square region‐of‐interest (ROI) was calculated using a script written in Python.

(1) $$OD=\mathrm{lo}g_{10}\frac{PV_{No}-PV_{Bg}}{PV_{F}-PV_{Bg}},$$

in which $PV_{F}$ is the mean pixel value of the scanned film within the ROI, which may correspond to either an irradiated or a nonirradiated film depending on the analysis being performed; $PV_{Bg}$ is the pixel value obtained by scanning an opaque sheet placed on the scanner bed to represent the scanner background level (dark signal) and does not correspond to a film sample; $PV_{No}$ is the pixel value obtained when no film sample is present on the scanner bed, representing the unattenuated transmitted light (Figure 1(h)). In order to minimize the impact of instantaneous fluctuations of the intensity of the scanner's light source, $PV_{No}$ was obtained for each scan by analyzing the scanned image and calculating the mean pixel value within a 100 pixel × 100 pixel ROI located outside the film area. The total scanned area (2320 × 739 pixels, corresponding to 19.6 × 6.3 cm^2^) was larger than the film dimensions (3.8 cm × 3.8 cm), allowing selection of a film‐free ROI to obtain the $PV_{No}$.

The net optical density (*Net OD*) of the film samples at a given time, t, was calculated through Equation (2) in which $OD_{0}$ is the OD of the unirradiated film (measured before irradiation) and $OD_{I}$ is the OD of the irradiated film scanned at time t.

(2) $$Net\;OD\left(t\right)=OD_{I}\left(t\right)-OD_{0}$$

The response curves (*Net OD* vs. dose) were obtained at several time points post‐irradiation (0.17 h, 6 h, 12 h, 24 h, 48 h, 120 h, 240 h, 730 h, and 1325 h) and fitted using an empirical function given in Equation (3):

(3) $$Net\;OD=A\times D+B\times D^{m}$$

in which A, B, and m are fitting parameters, and *D* is the delivered dose. The film sensitivity (*S*), defined as the derivative of Net OD with respect to dose, was calculated according to Equation (4).

(4) $$S=\frac{d\;Net\;OD}{dD}=A+B\times m\times D^{m-1}$$

To evaluate the effect of post‐irradiation readout time on film calibration and the resulting dosimetric errors, calibration curves were generated using the net OD values measured at the abovementioned selected post‐irradiation times using Equation (5).

(5) $$D=a\times Net\;OD+b\times Net\;OD^{n}$$

in which a, b, and n are fitting parameters. We used the calibration curve obtained at the 48‐h time point as a reference and numerically evaluated the dosimetric error when the net OD was increased by 1%.

## 3 RESULTS

**FIGURE 2:**
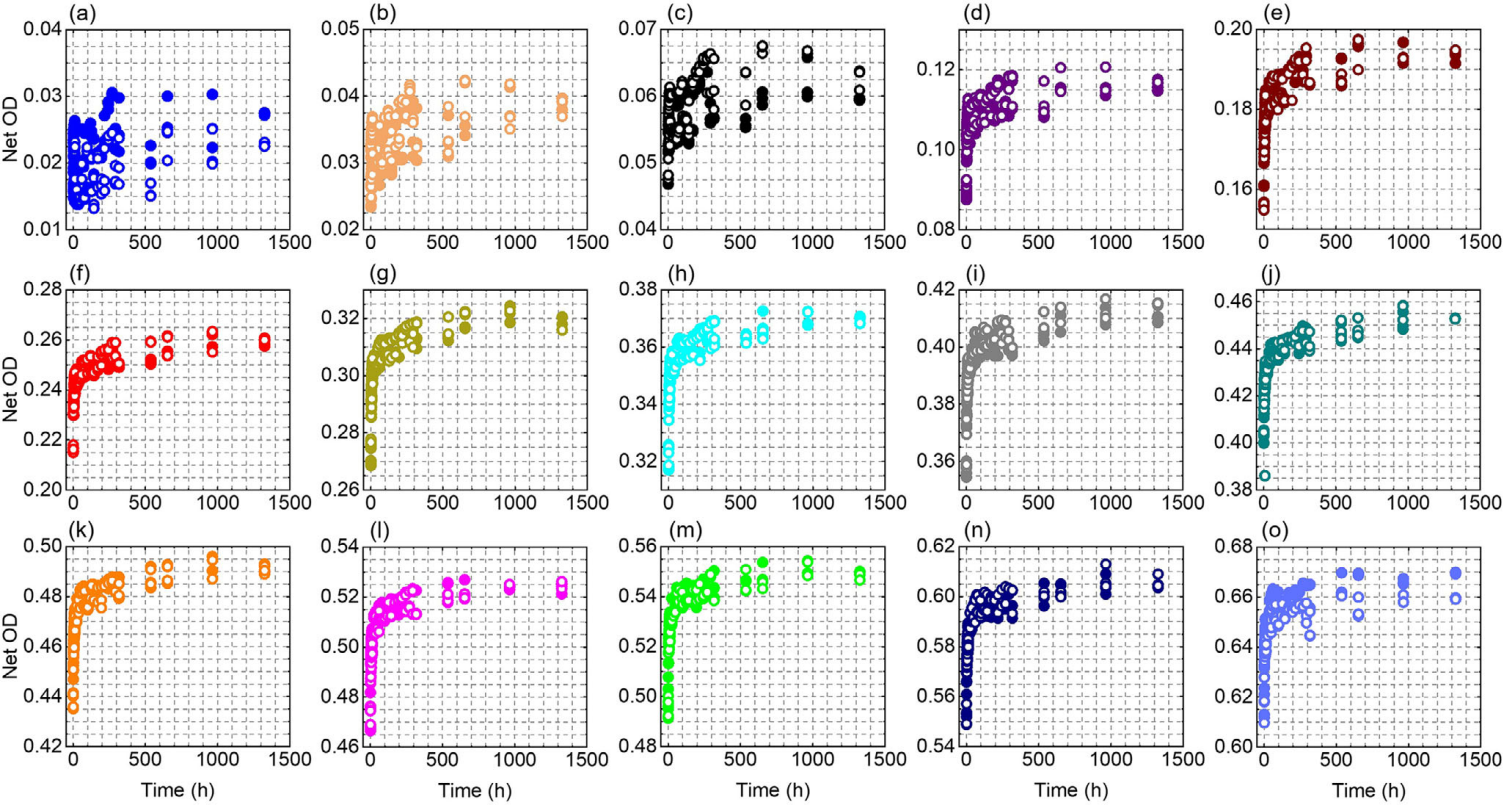
Net OD as a function of time, measured using the red color channel information, for two sets of films irradiated at various dose levels (a–o): 0.125, 0.25, 0.5, 1, 2, 3, 4, 5, 6, 7, 8, 9, 10, 12, and 15 Gy. Solid symbols are for Set 1 and hollow symbols are for Set 2.

**FIGURE 3:**
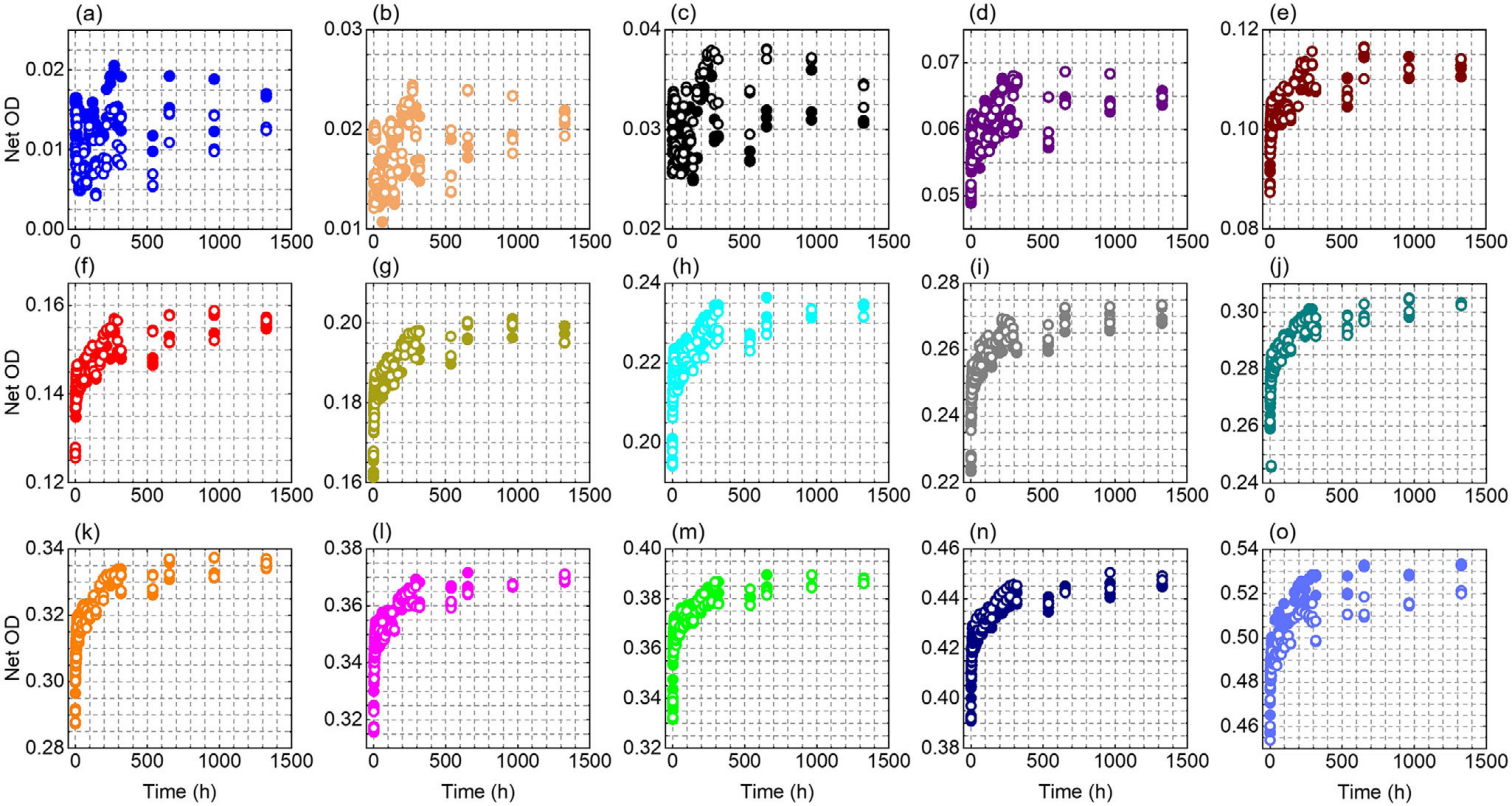
Net OD as a function of time, measured using the green color channel information, for two sets of films irradiated at various dose levels (a–o): 0.125, 0.25, 0.5, 1, 2, 3, 4, 5, 6, 7, 8, 9, 10, 12, and 15 Gy. Solid symbols are for Set 1 and hollow symbols are for Set 2.

**FIGURE 4:**
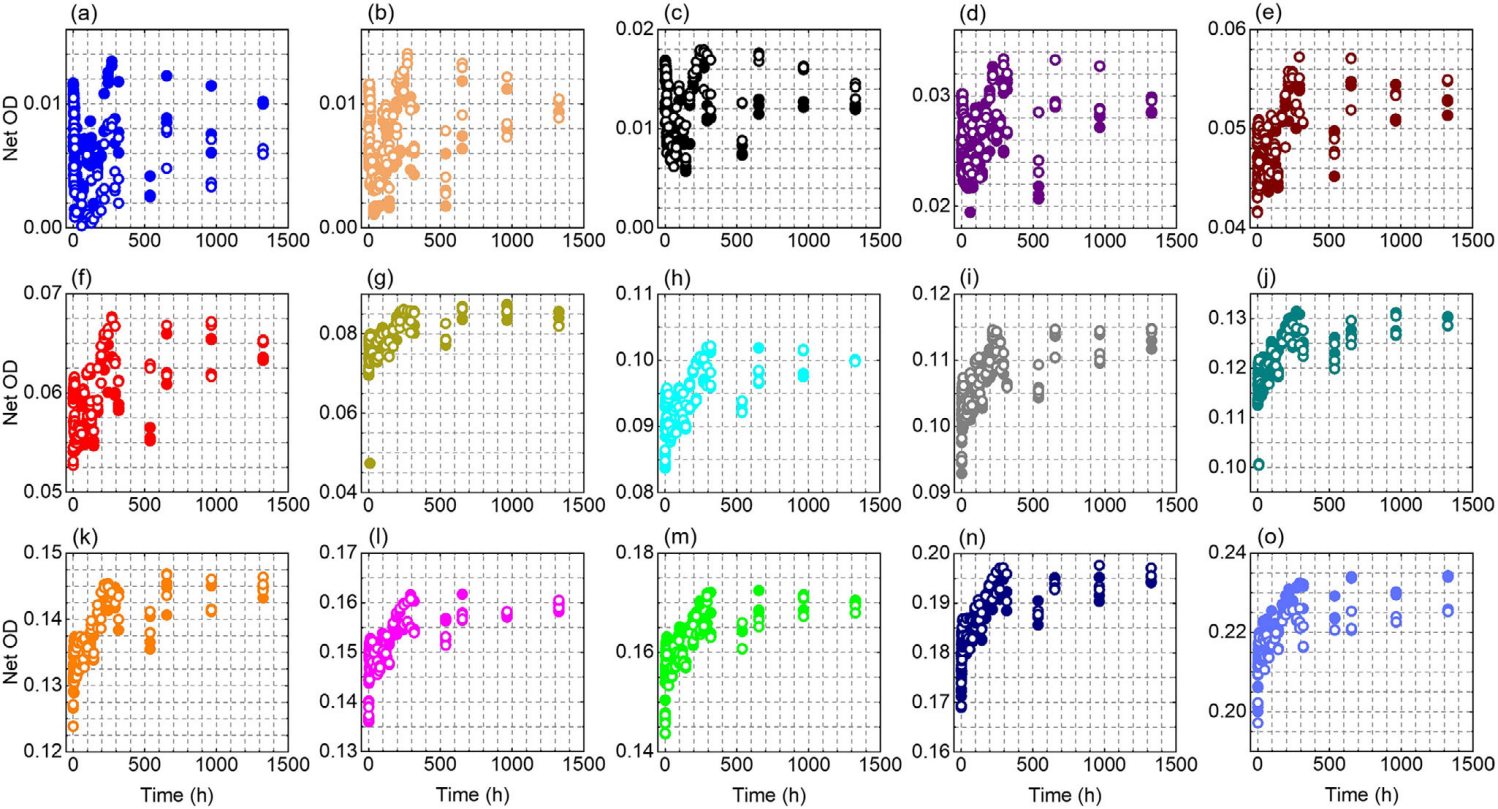
Net OD as a function of time, measured using the blue color channel information, for two sets of films irradiated at various dose levels (a–o): 0.125, 0.25, 0.5, 1, 2, 3, 4, 5, 6, 7, 8, 9, 10, 12, and 15 Gy. Solid symbols are for Set 1 and hollow symbols are for Set 2.

Figures 2, 3, 4 show the temporal evolution of the net OD measured from the red, green, and blue color channel, respectively, for the EBT4 films at the investigated dose levels (0.125–15 Gy). The red and green channel results become stable at doses ≥ 1 Gy and 2 Gy, respectively, manifested as closer grouping of the data points while the blue channel, which has a much weaker signal due to a much weaker radiation‐induced absorption at blue wavelengths,^32^, ^33^, ^34^ shows a clear and smooth response at doses ≥ 4 Gy. For doses above 1 Gy, the two independently irradiated film pieces show good agreement, with differences typically within about 1%–2% for the red channel and 1%–4% for the green channel at all measured times, while the blue channel shows a larger variation of approximately 5%–10%.

The temporal growth of the OD is evident in Figures 2, 3, 4 for all color channels. For example, in the red channel (for doses ≥1 Gy), the net OD grows at a time‐dependent and dose‐dependent rate; it grows rapidly immediately after irradiation reaching ∼92%–96% of the final net OD (i.e., net OD at ∼1325 h) within 12 h, and then continues to increase more slowly over the following weeks with a total additional growth of ∼8 % at 1 Gy and ∼4% at 15 Gy by the end of the measurement period (∼1325 h / ∼55 days). Similar trend was observed in other color channels.

**FIGURE 5:**
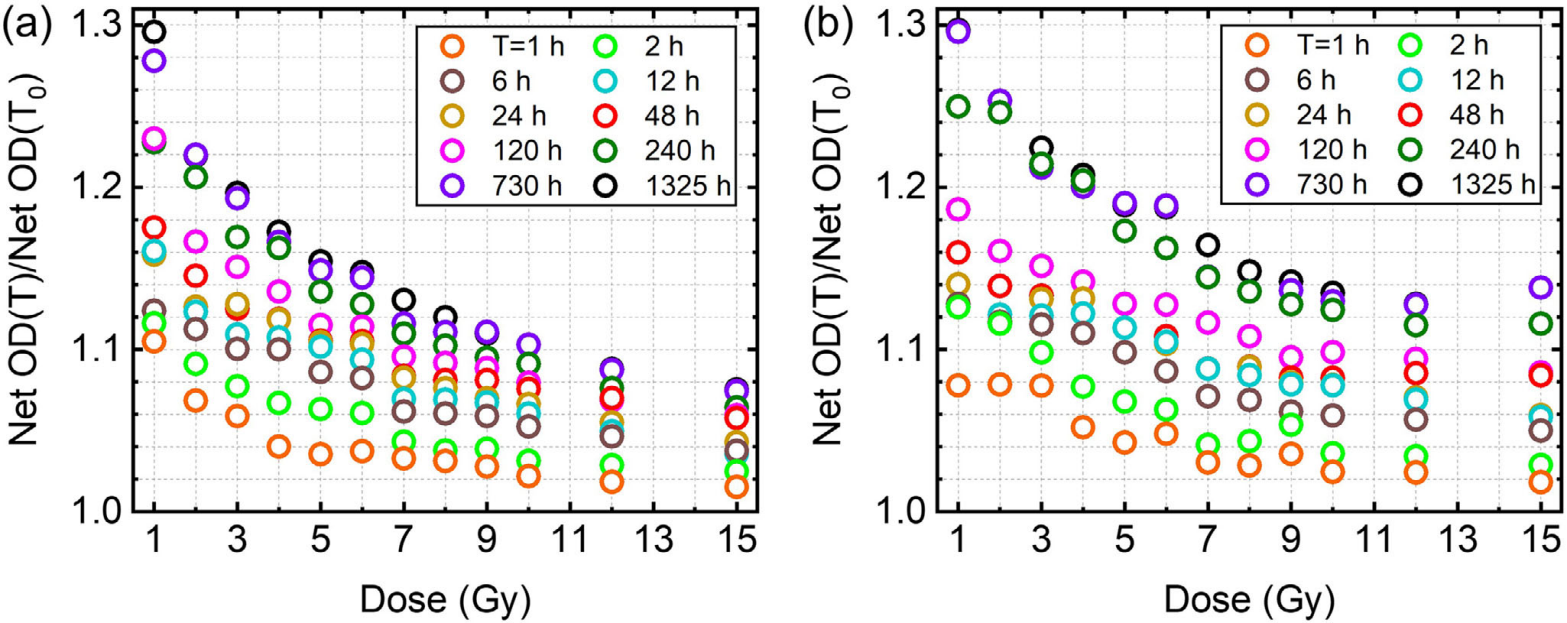
(a,b) The ratio of Net OD(T)/Net OD(T_0_) as a function of dose at several times (T = 1 h, 2 h, 6 h, 12 h, 24 h (1 day), 48 h (2 days), 120 h (5 days), 240 h (10 days), 730 h (∼1 month), and 1325 h (∼2 months) for the red and green color channel, respectively. T_0_ = 10 min.

To quantify the time‐dependency and dose‐dependency of the growth in the net OD, Figure 5(a,b) presents the ratio net OD(T)/net OD(T_0_) (i.e., at reference time T_0 _= 0.17 h) as a function of dose for selected readout times for the red and green color channel, respectively. In both panels, the ratio systematically decreases with increasing dose, indicating that post‐irradiation OD growth is more pronounced at low doses and becomes progressively weaker at higher doses. For the red channel (Figure 5(a)), representative post‐irradiation growth values show a clear dose‐dependence. At 1 Gy, the Net OD increases by approximately 12% at 6 h, 18% at 48 h, 28% at 730 h, and 30% at 1325 h. In contrast, at 15 Gy the growth is substantially smaller, reaching only about 4% at 6 h, 6% at 48 h, and 8% at both 730 h and 1325 h, indicating that post‐irradiation growth diminishes and approaches saturation at higher doses. Figure 5(b) shows the same overall trend as Figure 5(a) with slightly higher (∼1%–2%) Net OD(T)/Net OD(T_0_) values for the green channel, particularly at low doses and longer post‐irradiation times (≥120 h), indicating a somewhat stronger post‐irradiation growth under these conditions. At higher doses (approximately 10–15 Gy), the curves tend to flatten and converge, suggesting that the post‐irradiation growth approaches saturation and becomes largely dose‐independent. Overall, Figure 5 suggests that post‐irradiation growth is most pronounced at low doses or early times because more remining monomers are available in the active layer of the film to undergo polymerization, while the film response stabilizes more rapidly at higher doses. We did not perform the same analysis as Figure 5 for the blue channel due to more variations in the blue color channel data. Nevertheless, such approximate evaluation can be performed from Figure 4.

**FIGURE 6:**
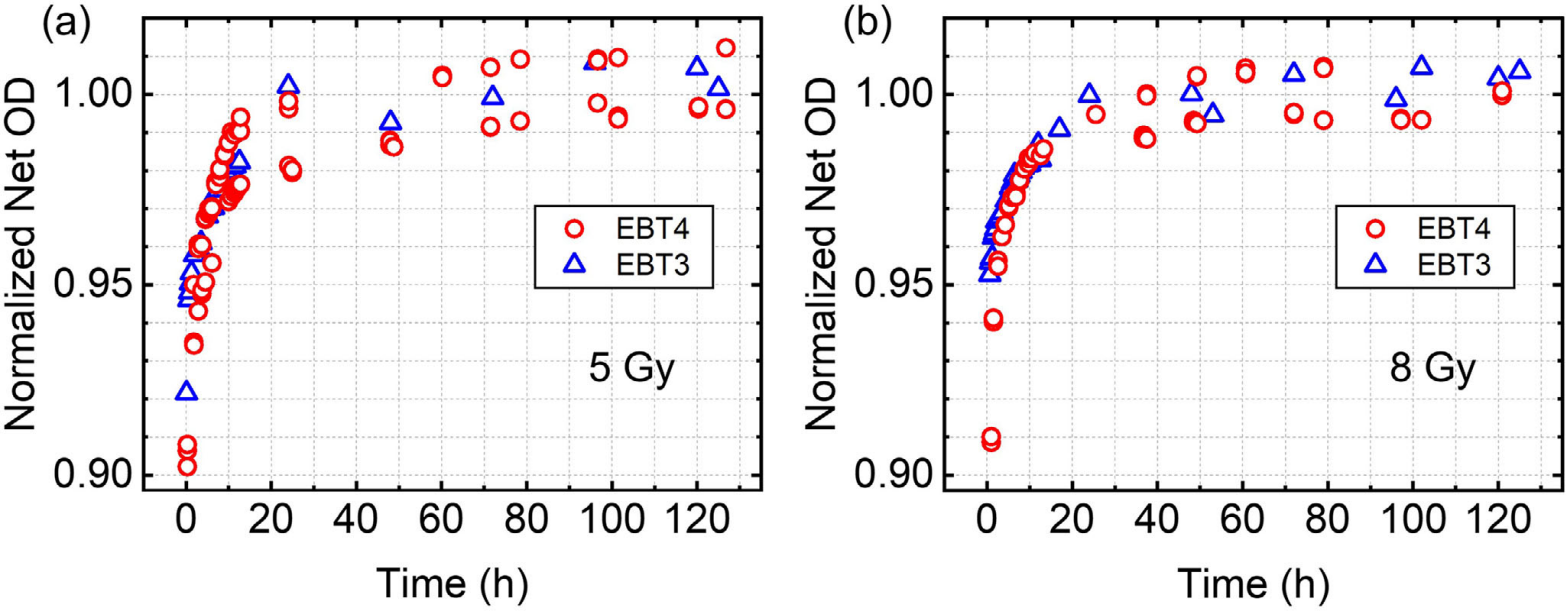
Comparison of normalized net optical density as a function of time between EBT3 and EBT4 films irradiated at (a) 5 Gy and (b) 8 Gy.

Figure 6(a,b) shows a comparison between EBT4 and EBT3 films irradiated at 5 Gy and 8 Gy, respectively. It can be seen that the temporal response of the EBT4 films is closely similar to that of EBT3 films reaching ∼98% of the final Net OD (at 120 h) within 12 h post‐irradiation followed by ∼1%–2% growth afterwards.

**FIGURE 7:**
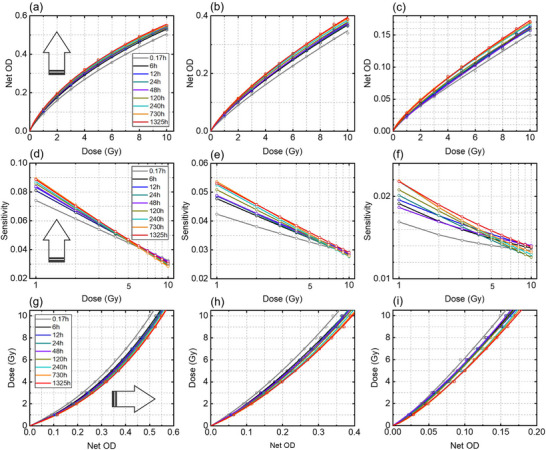
(a–c) Net OD as a function of dose at given times (0.17 h (10 min), 6 h, 12 h, 24 h, 48 h, 120 h, 240 h, 730 h (∼1 month), and 1325 h (∼2 months)) for the red, green, and blue color channels, respectively. (d–f) The corresponding sensitivity and (g–i) calibration curves. The arrows show which direction the plots are moving as time increases. Figure legends are the same in all panels.

The Net OD measured at multiple post‐irradiation times (0.17 h, 6 h, 12 h, 24 h, 48 h, 120 h, 240 h, 730 h, and 1325 h) is shown as a function of dose in Figures 7(a–c). The sensitivity, computed from Equation (4), is plotted versus dose in Figures 7(d‐f). Overall, the net OD increases monotonically with dose for all channels, while the sensitivity falls off as dose increases. In addition, the net OD and sensitivity both tend to increase with time after irradiation, reflecting continued post‐irradiation optical growth. Because the EBT4 absorption spectrum is dominated by red and green peaks,^14^ the blue channel exhibits the smallest signal and lowest sensitivity (Figure 7(f)). The corresponding calibration curves for the red, green and blue channels are given in Figures 7(g‐i), showing well‐behaved, monotonic dose‐response relationships that shift with readout time.

For the red channel (Figure 7 (a)), the net OD at 10 Gy increases approximately 10% due to post‐irradiation growth between 10 min to 1325 h. At lower doses (e.g., 3 Gy), the absolute net OD difference between early and late readout times is smaller but represents a larger relative change of ∼20%, indicating stronger time dependence at low doses. For the green channel (Figure 7(b)), the net OD at 10 Gy increases by ∼14% between 10 min and 1325 h; at 3 Gy, the corresponding difference is ∼22%. For the blue channel (Figure 7(b)), the net OD at 10 Gy increases by ∼18% between 10 min and 1325 h; at 3 Gy, the corresponding difference is ∼23%.

The sensitivity, defined as the slope of the response curve, decreases with increasing dose for all channels and readout times. For the red channel (Figure 7d), sensitivity drops from approximately 0.08 Gy^−^^1^ at 1 Gy to about 0.03 Gy^−^^1^ at 10 Gy, representing ∼60% decrease. For doses ≲6–7 Gy, at a given dose, sensitivity increases with time post‐irradiation, with the largest time‐dependent increase by ∼20% observed at 1 Gy. For the green channel (Figure 7(e)), sensitivity decreases from 0.040–0.055 Gy^−^^1^ at 1 Gy to ∼0.028 Gy^−^^1^ at 10 Gy, corresponding to a reduction of approximately ∼45%–50%. The blue channel in Figure 7(f) shows the lowest sensitivity, decreasing from roughly 0.016–0.022 Gy^−^^1^ at 1 Gy to ∼0.012–0.014 Gy^−^^1^ at 10 Gy, confirming its limited usefulness for low‐dose measurements. The calibration curves remain monotonic and smooth for all readout times and color channels, indicating stable dose‐response behavior. However, time‐dependent shifts are evident: at a given net OD, the inferred dose can differ significantly between early (e.g., 10 min) and later readout times. For practical comparisons we selected the 48‐h calibration for subsequent error estimation; an approximate error analysis indicates that a 1% relative error in net OD corresponds to ∼1%–1.5% relative error in estimated dose within the examined dose range, emphasizing that net OD differences due to inconsistent readout timing can produce clinically meaningful dose errors.

## 4 DISCUSSION

We showed that EBT4 RCFs continue to darken for several weeks after irradiation, with the most rapid changes occurring within the first 24 h. Overall, this behavior appears to be similar to what has been reported for EBT3 films. Dunn et al. observed that the red‐channel net OD (net OD at time *t* normalized to the net OD at 24 h) grew by about 4%–6% over the first 24 h post‐irradiation for EBT3 films across the 2–18 Gy range (6.1% at 2 Gy and 4.4% at 18 Gy).^25^ Liu et al.^24^ examined how the response of EBT3 films evolves after irradiation by comparing net OD measured shortly after exposure (∼5 min) with measurements taken 24 h later; over a wide dose range (1–40 Gy), they found that net OD continued to grow throughout the first 24 h in a dose‐dependent fashion. In particular, lower‐dose films showed larger relative changes: at 1 Gy, the red‐channel net OD at 5 min was ∼8% lower than the value at 24 h, decreasing to about 4% at 10 Gy, with progressively smaller differences at higher doses. In comparison, the EBT4 films investigated in our work exhibited a larger growth over the first 24 h, ranging from approximately 4% (at 15 Gy) to 13% (at 1 Gy) across the 1–15 Gy dose range. The difference between our EBT4 results and the published EBT3 data from Dunn and Liu is most noticeable at low doses (1–4 Gy), where the relative post‐irradiation growth is greatest and the film response is inherently less stable. Direct quantitative comparison between studies is challenging due to differences in scanner models and settings, film handling and storage conditions, as well as the film lots. Nevertheless, this behavior is consistent with the underlying polymerization process in RCFs: at lower doses, a greater fraction of monomer remains unreacted immediately after irradiation, allowing continued polymerization and proportionally larger darkening over time.

The differences between the EBT3 and EBT4 responses become much smaller as the films evolve toward a more stable state. Liu et al.^24^ showed that, for EBT3 films, only a modest additional increase in red‐channel net OD (on the order of ∼1%–3%) occurs after the first 24 h, even when measurements are extended to later time points. This behavior is similar to our observations for EBT4 films, where the net OD changed by only about 1%–2% between 24 and 48 h, with similarly small increases thereafter. These results suggest that although EBT4 films can exhibit slightly stronger early post‐irradiation darkening, particularly at low doses, the overall timescale required for the response to settle into a slower, quasi‐stable regime is comparable to that of EBT3.

In the long‐term (> 48 h) evaluation, EBT4 films continue to darken gradually for many weeks. In our measurements, the red‐channel net OD at 1 Gy increased by ∼10% between 48 h and ∼1325 h (∼55 days), while at 15 Gy the total increase over the same period was limited to ∼1.8%. This sustained growth beyond the first few days indicates that polymerization does not fully saturate within typical readout times, particularly at low doses. Comparable long‐term trends have been reported for other film models. Liu et al. measured EBT3 films up to 39 weeks post‐irradiation and found that, relative to 24‐h scans, net OD increased by ∼1.6% at 2 weeks and up to ∼4.3% at 39 weeks for doses ≥ 6 Gy, with larger relative changes at lower doses.^24^ Caprioli et al.^26^ reported long‐term transmittance decreases of up to ∼2.5% at 12.8 Gy in EBT3 films after 54 days, and showed that uncorrected long‐term drift could result in dose errors as large as ∼10%, which were reduced to ≤ 3% after applying a post‐irradiation correction model. Shameem et al.^35^ noted that film darkening in EBT4 films continues well beyond the first 48 h and can extend past 100 h, supporting our conclusion that extended monitoring (or time‐correction) is required for accurate long‐term dosimetry with EBT4. Palmer et al.^8^ directly quantified post‐exposure changes between ∼2 days and ∼19 days in EBT4 films, reporting an ∼1.9% pixel values increase in optical density at 10 Gy and ∼1.0% changes at 1 and 5 Gy over that interval. They noted that post‐exposure darkening continues beyond the first few days and recommend consistent scan timing to control this effect. Our extended dataset (∼55 days) complements Palmer et al.’s mid‐range observations by showing that higher doses approach practical saturation quickly while low doses (∼1 Gy) continue to evolve. Although Palmer et al. reported more pronounced long‐term darkening at higher doses, our data indicate that the relative long‐term increase is largest at the lower doses examined. This difference is consistent with polymerization kinetics, since lower doses leave a greater fraction of monomer unreacted immediately after irradiation, allowing continued polymer growth over time.

From a practical standpoint, similarity between temporal dynamics of EBT3 and EBT4 films means that the same dosimetric guidelines commonly used for EBT3 model can be applied to EBT4 model. In particular, calibration and measurement films should be scanned at the same elapsed time after irradiation, with a 24‐hour post‐irradiation delay serving as a practical and reliable choice for routine clinical work. The magnitude of the early‐time differences in ODs shows the risk of inconsistent readout timing. If films are scanned at mismatched times, especially at low doses, relatively small changes in net OD can translate into clinically meaningful dose errors. If workflow constraints necessitate earlier readout, time‐dependent calibration curves must be generated for the exact post‐irradiation interval used, using the same scanner, film lot, and storage conditions, with particular attention to low‐dose measurements where relative OD growth and dose sensitivity are greatest. In addition, when films are compared over extended time intervals of days to months, the continued slow post‐irradiation darkening should be explicitly accounted for, especially in applications requiring high absolute dose accuracy.

## 5 CONCLUSION

The response of EBT4 radiochromic films to radiation, quantified as net optical density, continuously increases with elapsed time after irradiation in all color channels. The rate of optical density growth is highest during the early post‐irradiation period and reduces progressively afterwards, with most of the optical density is developed within 12 h. This growth is dose‐dependent, with larger relative changes observed at lower doses and reduced growth at higher doses, consistent with saturation effects in film polymerization. The growth in net optical density for a given delivered dose with elapsed time after irradiation leads to time‐dependent calibration curves. If calibration and measurement films are scanned at different post‐irradiation times, this temporal evolution can introduce significant errors in dose estimation. Therefore, calibration and test films must be scanned at the same elapsed time after irradiation. The temporal response of EBT4 films was found to be similar to that of EBT3 films, hence, in practice, as with EBT3 films, a post‐irradiation waiting period of approximately 24 h is sufficient to minimize the impact of temporal changes in optical density and is recommended for routine EBT4 film dosimetry.

## AUTHOR CONTRIBUTIONS

**Arash Darafsheh**: Conceptualization; methodology; analysis; investigation; writing—original draft; writing—review and editing; visualization. **Ananya Parimi**: Methodology. **Megha Goddu**: Analysis; writing—review and editing. **Hamid Ghaznavi**: Analysis; writing—review and editing. **Aditi Purushothaman**: Analysis. **S. Murty Goddu**: Methodology; writing—review and editing. All authors have read and approved the final version of the paper.

## CONFLICT OF INTEREST STATEMENT

The authors declare no conflicts of interest.
